# Pilot Trial of the Reboot Online Program: An Internet-Delivered, Multidisciplinary Pain Management Program for Chronic Pain

**DOI:** 10.1155/2018/9634727

**Published:** 2018-09-05

**Authors:** Regina Schultz, Jessica Smith, Jill M. Newby, Tania Gardner, Christine T. Shiner, Gavin Andrews, Steven G. Faux

**Affiliations:** ^1^Department of Pain Medicine, St Vincent's Hospital, Sydney, NSW, Australia; ^2^Clinical Research Unit for Anxiety and Depression (CRUfAD), School of Psychiatry, St Vincent's Hospital, University of New South Wales, Sydney, NSW, Australia; ^3^School of Psychology, University of New South Wales, Sydney, Australia; ^4^St Vincent's Clinical School, University of New South Wales, Sydney, Australia

## Abstract

**Objectives:**

Chronic pain causes significant disability and psychological distress, but barriers often prevent people with pain from engaging in traditional face-to-face pain management programs. Accessible, feasible, and effective alternative treatment options are needed.

**Methods:**

A prospective, feasibility pilot study was conducted to trial a novel, multidisciplinary online pain management program: the “Reboot Online” program. Twenty participants experiencing pain of at least three months duration were recruited. All participants were enrolled in the “Reboot Online” program, consisting of eight online lessons completed over 16 weeks. Lessons incorporated multidisciplinary input from medical pain specialists, physiotherapists, and psychologists. Participants were assessed at pretreatment, posttreatment, and follow-up using a suite of outcome measures examining pain, disability, catastrophising, self-efficacy, mood, and psychological distress.

**Results:**

13 participants completed the program (65% adherence). Following treatment, the participants had significantly improved scores on measures of pain-related disability, self-efficacy, catastrophising thoughts, acceptance of pain, symptoms of depression, and general psychological distress. These findings were retained at three months posttreatment. Participants also reported high levels of acceptability and satisfaction with the program.

**Discussion:**

This study provides pilot evidence for the feasibility, acceptability, and effectiveness of an online, multidisciplinary pain program: “Reboot Online.” Future investigations will focus on conducting a randomised controlled trial of this innovative and promising treatment for chronic pain. This trial is registered with ACTRN12615000660583.

## 1. Introduction

Chronic pain is a multifaceted health problem with considerable burden at the individual and global level [1–3]. Multidisciplinary pain management programs (MDPPs) are the accepted best practice for chronic pain management [4, 5]. These are face-to-face treatment programs, designed to reduce disability and suffering and typically delivered in a group-based setting by a multidisciplinary pain team. These programs generally require participants to engage in face-to-face sessions of moderate (30–60 hrs) to high (>60 hours) intensity [6] and are mostly based in urban outpatient treatment facilities. This common delivery method typically restricts access for those living in rural areas, those with work and family commitments, and/or those with disabilities which prevent travel and/or face-to-face contact. Often, access to MDPPs can be further restricted by long waiting times for specialist services and the stigma surrounding chronic pain. A viable and effective Internet-based pain management program could improve access to evidence-based multidisciplinary treatment for people with chronic pain who cannot access face-to-face services.

In recent years, a strong evidence base has developed for the efficacy of Internet-based psychological treatments, especially those based on cognitive behavioural therapy (CBT) principles, to treat a wide range of mental health conditions including depression and anxiety disorders [7–9]. Moreover, several Internet-based treatment programs for chronic pain have been developed, with burgeoning evidence indicating efficacy in reducing disability, mood disturbance, and improving perceived self-efficacy [10–14]. However, the efficacy of these programs is variable due to methodological inconsistencies in the literature, including different outcome measures, key program content, types of control groups used, and clinician involvement [15]. A few meta-analyses and systematic reviews have been completed thus far and report varying effect sizes. Bender et al. [16] reported significant improvement on measures of pain intensity, depression, anxiety, and functional measures for participants who completed online pain management programs compared to controls; however, it is difficult to compare these results with other reviews because this paper did not report effect sizes. Another systematic review reported effect sizes ranging from small to large for pain intensity measures (0.20–0.85); however, most were in the small to moderate range with only one (out of 19) reporting a large effect size [15]. A consequent systematic review duplicated the small to moderate effect sizes for pain intensity scores (−0.35 to −0.16), measures of catastrophising (range −0.32 to −0.26), depression (range −0.18 to −0.14), and functional interference (−0.35 to −0.16; [14]). Therefore, given the typically small to moderate effect sizes reported of Internet-based pain management programs, it is warranted to explore the viability of developing a novel program to improve treatment efficacy.

Clinical practice guidelines for chronic pain management promote a multidisciplinary approach which includes a self-management framework with cognitive behavioural techniques, graded activities and exposure, methods to improve acceptance and cognitive flexibility, skills training, education, and notably physical exercise [5, 17–19]. In addition, the literature suggests that although no specific exercise is most beneficial, a graded approach to physical exercise is a key component of chronic pain management in reducing disability, especially for low back pain [20, 21]. Strategies which include an individualised approach, refresher sessions, or the addition of audio or video tapes can improve exercise adherence [20].

Review of the content of the existing Internet-based pain management programs shows the programs include some of these components, in different configurations, but none include all. More specifically, none provided a structured physical exercise section. Therefore, given the demonstrated relative efficacy of existing online pain management programs, the “Reboot Online” program was developed to provide a comprehensive multidisciplinary approach with all the components, with particular attention to incorporating a physical exercise component. Thus, the “Reboot Online” program, to our knowledge, is the only online pain program which includes a full multidisciplinary approach, incorporating psychoeducation, psychological skills, and a tailored graduated physical activity program. Another advantage is that the program can be monitored and used to supplement treatment plans by any member of the multidisciplinary pain team including doctors, physiotherapists, social workers, psychologists, or practice nurses. Consequently, the “Reboot Online” program provides a complete multidisciplinary approach.

The “Reboot Online” program was developed via a unique collaboration between clinical experts in chronic pain management (Department of Pain Medicine, St Vincent's Hospital, Sydney, Australia) and leading researchers in the development of Internet-based CBT (iCBT) programs (Clinical Research Unit for Anxiety and Depression, University of NSW, St Vincent's Hospital, Sydney, Australia; CRUfAD). It is closely modelled on the Reboot Pain Management program, a face-to-face MDPP developed at St Vincent's Hospital, Sydney, Australia. This group-based MDPP runs for 6 hours per day, one day per week, for 10 weeks and is located on-site at St Vincent's Hospital, Sydney. Preliminary data suggest that this program is effective in improving measures of pain, pain disability, and psychological measures of distress [22]. In addition, the “Reboot Online” program was modelled on existing iCBT programs successfully developed at CRUfAD to treat anxiety and depression [8, 23, 24].

This paper describes a pilot study conducted to explore the feasibility, acceptability, and outcomes of the novel “Reboot Online” program for chronic pain.

## 2. Materials and Methods

### 2.1. Participants

Participants with self-identified pain were recruited via hospital and Facebook advertisements and the “Virtual Clinic” website (www.virtulclinic.org.au), an online research portal of CRUfAD. Inclusion criteria were as follows: aged ≥18 years; self-reported experience of pain for ≥3 months; resident of Australia; and prepared to provide participant and general practitioner contact details. In addition, participants were required to have a phone, a computer with Internet access, be on stable doses of medications for at least three months, and have had their pain assessed by a physician within the last three months. Exclusion criteria included: an inability to communicate in English; a current diagnosis of psychosis, bipolar disorder, substance abuse or dependence; or being actively suicidal. Participants with a pain-related surgical intervention/treatment scheduled in the next six months or participants who had completed a multidisciplinary pain management program within the prior six months were also excluded.

**Figure 1:**
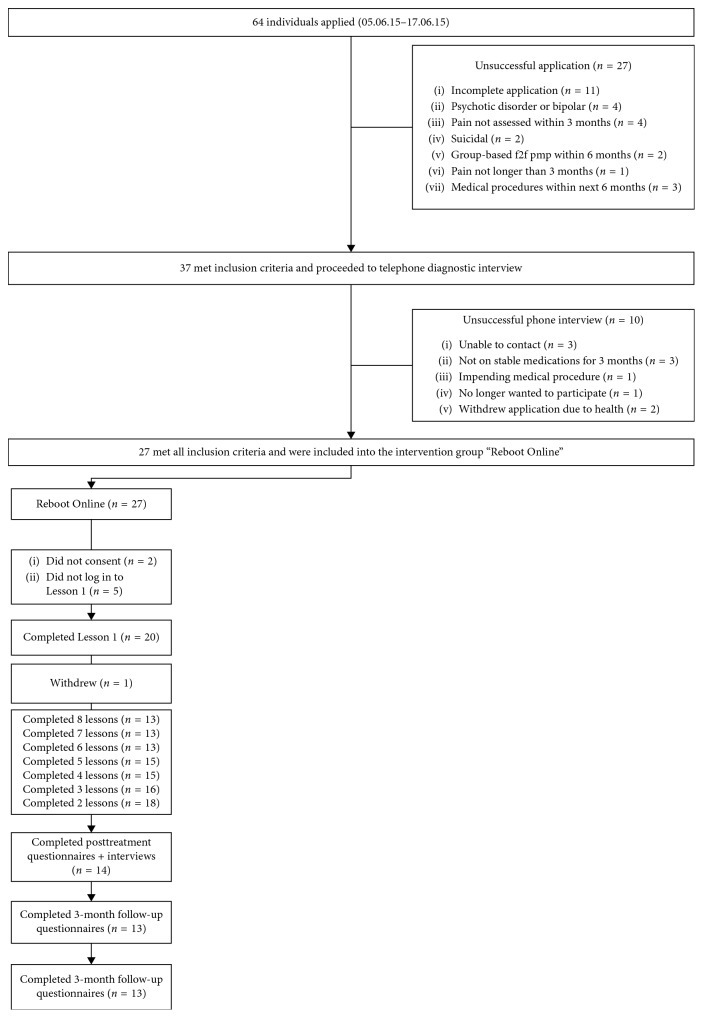
Participant flow for recruitment and adherence.

Potential participants applied online, and screening was conducted in two phases, first via an online questionnaire [25] and subsequently via a structured phone interview for those who passed online screening (Figure 1). Phone interviews were conducted by a trained clinician to confirm the diagnosis of chronic pain and assess for the presence of a major depressive disorder. Eligible participants who met all inclusion and no exclusion criteria following both screening processes were invited to participate in the trial. All provided informed electronic consent prior to commencing the program. A letter was also sent to each participant's GP/pain physician informing them of the participant's enrolment into the trial.

Sixty-four applicants were screened, 27 met all inclusion criteria, and 20 eligible participants were enrolled into the study (Figure 1). Baseline data from all 20 participants are included in the analyses, while posttreatment and follow-up data are available for 14 and 13 participants, respectively. This study was approved by the Human Research Ethics Committee of St Vincent's Hospital Sydney, Australia (HREC/15/SVH/32), and conducted in accordance with the Declaration of Helsinki. The trial was prospectively registered on the Australian and New Zealand Clinical Trials Registry (ACTRN12615000660583).

### 2.2. Treatment Program

The “Reboot Online” program consists of eight online lessons, to be completed over 16 weeks by the participant. The course was accessed via the Virtual Clinic website, with the release of content staged throughout the course duration (one new lesson released every two weeks). Lesson content is centred around an illustrated storyline that depicts a fictional character with chronic pain. Each lesson follows this character as they engage with multidisciplinary treatment to learn about and manage their chronic pain. Lesson content was focussed on (1) an introduction to the chronic pain model, (2) goal setting and acceptance, (3) movement, pacing, and daily activity scheduling, (4) monitoring and recognising unhelpful thoughts, (5) mood and pain and working with unhelpful thoughts, (6) stress and sleep management, (7) communication and relationships, and (8) managing flare-ups. With each nominated lesson, the participant has access to a downloadable lesson summary with practical homework exercises, related educational videos, a Tai Chi video, relaxation audio files, and an “at home” exercise program. Participants were only able to progress to the next lesson if they had accessed each component of the current lesson.

The core course material incorporates content developed with multidisciplinary expertise from medical, psychiatric, occupational therapy, physiotherapy, and psychology pain specialists. Unique to this program is the inclusion of a graded exercise program, accessed through the “movement station” and tailored to the needs of each participant. This incorporates four key components, namely, strength, stability, flexibility, and cardiofitness. For the strength, stability, and flexibility components, a prescribed exercise program including video demonstrations was selected, at one of four intensity levels. Progression of exercise was self-determined by the participant, although guidelines were provided that outlined the suggested competency level required in each exercise before progression. For the cardiofitness component, participants self-selected an appropriate activity to complete. Progression through to the next lesson was conditional on the participant accessing the movement station. A graded Tai Chi (Yang style) program incorporating demonstration videos and instructions was similarly provided to participants at the beginning of the program, to be completed over the course duration. Four relaxation audio files and instructions on how to practise relaxation and the benefits of relaxation for chronic pain management were included in the online material.

To supplement the core course material, participants had access to nine educational videos providing information on related topics including nutrition, common pain medications, the role of medical imaging in pain management, the nature of chronic pain, activities of daily living, and ergonomics. Viewing was encouraged, but not compulsory.

Participants were informed that during the course of the program, they had email access to a multidisciplinary team including a psychiatrist, pain specialist (medical), clinical psychologist, and physiotherapist from the trial team, who were available to respond to participant questions and concerns. Participants also received email and/or phone contact from an allied health technician (JS) until they completed lesson 2.

Of note, a participatory design process was used to include consumer feedback into the design of the “Reboot Online” program. Specifically, a focus group of service users (*n*=9) was convened to provide feedback on the format, language, content, and structure of the program. The service users were provided with Lesson 1 from the program approximately one month before the pilot trial was launched to ensure sufficient time to incorporate appropriate changes into the program design.

### 2.3. Outcome Measures

Participants were formally assessed via a suite of measures at four time points: pretreatment, midtreatment, posttreatment and at three-month follow-up. Data from three time points (pretreatment, posttreatment, and follow-up) will be the focus of the current paper. As per the Virtual Clinic guidelines, prior to the start of each lesson, participants were required to complete the Kessler 10-item Psychological Distress Scale (K10) [26] to monitor any increase in psychological distress; participants were contacted if their scores significantly deteriorated.

#### 2.3.1. Primary Outcome Measures

The two primary outcomes of interest were as follows:

- The Pain Self-Efficacy Questionnaire (PESQ) [27–30], a 17-item self-report questionnaire designed to assess the participant's confidence to perform activities while experiencing pain.
- The Brief Pain Inventory (BPI) [31, 32], a 9-item questionnaire designed to measure the severity of pain and the interference of pain on daily function.

#### 2.3.2. Secondary Outcome Measures

- The Pain Catastrophising Scale (PCS) [33, 34], a 13-item self-report measure of catastrophic thoughts related to chronic pain.
- Tampa Scale of Kinesiophobia (TSK) [35, 36], a 17-item checklist which measures fear and avoidance of movement.
- Pain Disability Index (PDI) [37, 38], which measures the impact of pain on the participant's confidence to perform activities while experiencing pain.
- Chronic Pain Acceptance Questionnaire (CPAQ) [39, 40], which examines the participant's acceptance of chronic pain.
- The Depression Anxiety and Stress Scale-21 (DASS-21) [41, 42], a measure of the negative emotional states of depression, anxiety, and stress via the frequency of associated symptoms. Depression, anxiety, and stress subscores were used for analyses.
- The Kessler 10-item Psychological Stress Scale (K10) [26, 43], a self-reported measure of overall psychological distress.
- The Patient Health Questionnaire-9 (PHQ-9) [25], for symptoms of depression.

Given the feasibility nature of this study, data on acceptability of the program were also collected, via the following measures:

- Time spent reading lessons, reported by each participant as a measure of engagement with the program.
- Treatment satisfaction, via completion of an 18-item satisfaction questionnaire posttreatment. Each item was scored on a 0 to 5 Likert scale.
- Adherence rates, for completion of each lesson and the overall program.

### 2.4. Statistical Analyses

Linear mixed models were used to investigate changes in primary and secondary outcome measures from pretreatment to posttreatment, and pretreatment to 3-month follow-up. A MIXED procedure with a random intercept for subject was used, as in iCBT previous studies [44]. Mixed models are capable of estimating parameters for repeated measures studies with missing data via maximum likelihood estimation, thus were appropriate for this study [45]. For each outcome, time was treated as a categorical variable, and an identity covariance structure was specified to model the covariance structure of the random intercept. Initial model building focussed on the selection of the most appropriate covariance structure for the residual correlation matrix. Model fit indices and inspection of the variance-covariance matrix supported the selection of the identity covariance structure for each of the outcome measures. Effect sizes (Hedges *g*, adjusted for sample size) were calculated to determine the size of the within-group reduction between pretreatment to posttreatment, and pretreatment to 3-month follow-up. All analyses were implemented in SPSS v24, and differences where considered significant when *p* < 0.05.

## 3. Results

### 3.1. Participant Demography

20 participants were enrolled in the “Reboot Online” program, of whom 19 were female (95%) and mean age was 47.5 years. 35% of participants (*n*=7) reported to reside in a major city, 30% in outer regional areas (*n*=6), 20% in remote areas (*n*=4), and 15% in inner regional zones (*n*=3). The majority of participants were not in paid employment (75%; *n*=15); 30% were registered as disabled (*n*=6), 20% were retired (*n*=4), 15% were at-home parents (*n*=3), and 10% were seeking work (*n*=2). The remaining 25% were in full-time or part-time employment.

The majority of participants had experienced pain for longer than 5 years (65%) and had never participated in a MDPP before (80%). All but one participant (*n*=19, 95%) reported that their pain was always present, they did not have an active compensation claim, and that their pain affected the number of hours they were able to work. Half the participants (*n*=10) reported that their main pain started from no obvious cause, with the remaining describing their pain arising from illness (25%, *n*=5), injury (15%; *n*=3), and other causes (10%; *n*=2). Apart from pain, the sample was relatively healthy, with nearly all participants denying a previous diagnosis of lung disease (*n*=20), cancer, heart, stomach or kidney disease (*n*=19), stroke, diabetes or blood diseases (*n*=18), or high blood pressure (*n*=17). In terms of psychological disorders, just over half the sample described a previous self-reported diagnosis of depression (55%; *n*=11) and 25% (*n*=5) reported a diagnosis of post-traumatic stress disorder (see Table 1 for detailed descriptive data).

### 3.2. Adherence and Engagement

All 20 participants completed lesson 1, 18 completed lesson 2, 16 completed lesson 3, 15 completed lessons 4 and 5, and 13 completed all 8 lessons. This represents a 65% completion rate for the program in its entirety. Participants spent an average of 39–80 minutes completing each lesson and 89–156 minutes practising the relevant skills. However, this included large intersubject variability, with self-reported time spent reading the lessons ranging from 10 to 240 minutes and between 0 and 1200 minutes for practising the skills. During the three-month follow-up period, the participants reported that they spent on average 10.6 hours (637.5 minutes) practising skills they had learnt in the program. Regarding clinician contact, the allied health technician (JS) spent an average of 40.5 minutes (*SD *= 33.46, range 6–121) emailing and calling each participant during the treatment course (including the follow-up period), and this included relaying responses to individual participant's queries from the multidisciplinary team.

### 3.3. Primary and Secondary Outcomes at Posttreatment

**Figure 2:**
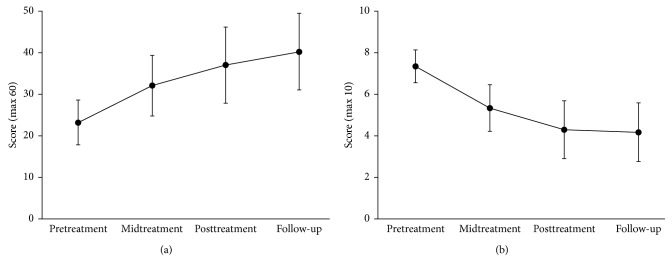
Participant improvement on primary outcome measures. (a) The Pain Self-efficacy Questionnaire (PSEQ), where higher scores indicate greater self-efficacy. (b) The Brief Pain Inventory (BPI)-Interference Score, where lower scores indicate less pain interference. Data are presented as mean and 95% confidence intervals.

Table 2 describes the summary statistics, estimated marginal means, and the linear mixed model results for the primary and secondary outcomes at pre- and posttreatment and follow-up. For the primary outcome measures, a statistically significant change was observed between pre- and posttreatment for the PSEQ and for the BPI interference scale, with large effect sizes observed for both (Table 2 and Figure 2). Among secondary measures, significant differences were observed on the CPAQ, PCS, K10, and the depression subscale of the DASS-21. Large effect sizes were observed for nearly all secondary outcome measures (range *g* = 0.82 to 1.46), except the TSK, DASS-A, and DASS-S score which showed moderate effect sizes (*g *= 0.54–0.66; Table 2).

### 3.4. Primary and Secondary Outcomes at Three-Month Follow-Up

The primary and most of the secondary outcome measures, demonstrated a statistically significant difference between the pretreatment and 3-month follow-up scores (Table 2). The large effect sizes were retained at 3-month follow-up; the primary outcome measures demonstrated large effect sizes (*g *= 1.24 to 1.53), and large effect sizes were also observed for all the secondary outcome measures (range *g *= 1.01 to 1.30), except the TSK, DASS-A, and DASS-D scores which retained a moderate effect size (range *g *= 0.66–0.76).

### 3.5. Participant Satisfaction

Overall, participants were satisfied with the program, 8 reported to be “very satisfied” and 5 “mostly satisfied”. The majority reported that their confidence to manage their pain had improved (“significantly increased,” *n*=7; “increased,” *n*=5; “no change,” *n*=2). Moreover, nearly all the participants rated their confidence in the program teaching them pain management techniques was at least 7/10, with 8 participants rating their confidence as 10/10. When asked to rate the quality of their contact with the clinical team, all participants rated this as either excellent (*n*=10) or good (*n*=3). Similarly, the participants rated the quality of the materials in the program as either excellent (*n*=7) or good (*n*=6).

Regarding individual components of the program, the lessons, lesson summaries, and relaxation recordings were rated by all participants as either extremely important or important. Most other components were also rated as extremely important or important, with only a few participants rating the movement station (*n*=2), the resources section (*n*=1), automatic emails (*n*=1), and clinical support from the team (*n*=1) as not very important. Participant feedback identified some areas for improvement to the program. Specifically, approximately half the participants (*n*=7) thought there was not enough time to complete the program and only six participants described themselves as relating well to the fictional online character. This feedback will be incorporated into future modification of the program.

## 4. Discussion

To our knowledge, “Reboot Online” represents the first comprehensive, Internet-based, multidisciplinary treatment program for chronic pain management. The structure and content of this program have been closely modelled on established face-to-face treatment programs for chronic pain, making it the first online resource to mirror face-to-face services and translate them to an Internet-based platform. The findings of the current pilot study show that following completion of the “Reboot Online” program, participants' scores significantly improved on measures assessing their ability to manage and accept their chronic pain. In addition, there was a significant reduction in scores on measures examining perceived level of disability, interference from pain, and catastrophic thoughts relating to pain. An accompanying reduction in symptoms of general psychological distress and depression were also observed. These findings were evident immediately after completion of the program and were largely retained at three months. In comparison to the typically small to moderate effect sizes previously reported in the literature [14, 15], the current results demonstrate large effect sizes on the majority of measures posttreatment (range: 0.81–1.46) and moderate effect sizes on the remaining (range: 0.54–0.66). Even compared to more efficacious Internet-based pain programs, the current results are at least comparable and on some measures more favourable [12]. This suggests that the comprehensive multidisciplinary approach delivered by “Reboot Online” is a feasible treatment option. Most notably, effect sizes even increased at follow-up for some measures, and this differs from established interventions where improvement often decays over time [46], suggesting that this novel intervention may represent not only a feasible, but also sustainable treatment approach for the ongoing management of chronic pain.

Our data demonstrate that there was a high level of engagement with the program, with participants spending an average of at least 30 minutes reading the lessons and nearly 1.5 hours applying and utilising newly acquired skills in their everyday life. This suggests that the program was engaging for its target population and that it assisted people to incorporate pain management strategies into their lifestyle. Generally, participants found the different multidisciplinary aspects of the program to be useful and important and reported that their confidence to use pain management techniques and to manage their pain improved following the program. The moderate adherence rate suggests that not all participants could sustain their engagement with the program; however, the overall high satisfaction rate, which is comparable to similar online pain management programs [12], suggests that “Reboot Online” was an acceptable and well-received intervention.

The demographic characteristics of the current sample are similar in terms of the average age and duration of pain compared with reported participant demographics from online pain management programs and face-to-face multi-disciplinary pain management clinics [14, 47, 48]. The baseline scores of the current sample on several questionnaires (DASS-D; DASS-A; DASS-S; PHQ-9; PSEQ; TSK) were comparable to those reported by participants accessing Australian and Canadian face-to-face multidisciplinary pain clinics [47, 48]. However, the current sample differed to the existing literature regarding the percentage of employed participants, with more participants actively employed in the previously reported literature (range: 30.4%–67%), compared to the current sample (25%) [47, 48]. In addition, the gender distribution was not comparable, with previous literature typically reporting fewer female participants (range: 57.4%–71.1%) than the current sample (95%); however, given the small sample size, caution should be used in making conclusions about the influence of the gender distribution on the present results [47, 48]. Therefore, the demographic characteristics and baseline scores on several of the primary and secondary outcome measures are comparable to participants accessing other online pain programs and face-to-face multidisciplinary pain clinics.

### 4.1. Methodological Considerations

There are some limitations of the current study, most notably the adherence rate was reasonable but not high; 35% of the initial participants did not complete all the lessons. This is comparable to the mean withdrawal rate (27.4%) reported in a systematic review of Internet-based pain management programs (range: 5.7% to 58.9%), with more than half reporting baseline differences between those who completed and withdrew from the studies [16]. However, the duration of “Reboot Online” (16 weeks), is longer compared to other online pain programs (8 weeks) [12] and given the added requirement for participants to access the graded exercise program for each lesson, the adherence rate is notable compared to programs with less onerous time and commitment requirements. Also, many of the participants who completed the trial reported that they did not relate well with the fictional character at the centre of the program. It is difficult to determine why some participants did not complete the lessons or engage well with this character. Perhaps this was because there is limited capacity to individually tailor the program content, skills, and techniques provided in an online format, as compared to face-to-face programs.

Given the small sample size, definitive conclusions about program efficacy cannot be drawn from this pilot study, rather, it should be used to inform the design of a larger, more rigorous clinical trial. The small sample and substantial missing data may have also led to an overestimation of treatment effects. Being exploratory in nature, this pilot study also examined a wide variety of outcome variables. While this may limit statistical power, here it was an important scoping exercise to determine the most pertinent measures to include in future analyses.

For each lesson, it was compulsory for the participant to access the movement station; however, limitations in the software application meant that information on how many times or how long the participants watched the exercise videos could not be recorded. Thus, there is not a clear indication of the participants' engagement with the graded exercise program. Furthermore, no direct measure of physical activity was included in the present study, due to a lack of consensus on a “gold-standard” measure, in addition to the logistical challenge of obtaining physical activity data from subjects who are engaging remotely in an online program. Given that the comprehensive multidisciplinary approach adopted by the “Reboot Online” program is novel and particularly the inclusion of a graduated exercise program, this may be of interest in future studies.

“Reboot Online” is likely to appeal to a broad section of the chronic pain population because the delivery method of the course is sufficiently convenient for those unable to attend a hospital or a face-to-face pain management centre due to family, work, health, or transport issues. Evidence for the optimal management of chronic pain supports treatment by a multidisciplinary pain team [5, 19]; however, these are rarely located in rural and remote areas in Australia. An online program represents a novel mode of service delivery, especially for those unable to face-to-face programs, thus improving equity of access to state-of-the-art multidisciplinary treatment. This is supported by the demographic profile of the current sample, with the majority of participants not residing within a major city. Finally, many people suffering from chronic nonmalignant pain have a need for education and comprehension of the problem as indicated in the Australian national pain strategy [49]. While existing online resources such as the Australian Agency for Clinical Innovation (ACI) Pain Network website may offer readers access to information [50], the “Reboot Online” MDPP can also offer effective treatment to those who have gained an understanding of their problem and the opportunity to learn relevant skills to manage their pain. The program will also benefit a range of clinicians because the design of the program allows it to be monitored by any member of the multidisciplinary pain team including doctors, physiotherapists, social workers, psychologists, or practice nurses. In addition, solo practitioners such as GPs, individual psychologists, or physiotherapists could use the program to supplement their treatment plan for patients.

## 5. Conclusions

The results of this pilot trial demonstrate that the novel “Reboot Online” program is a feasible and acceptable treatment program for the chronic pain population. Preliminary data suggest that this Internet-delivered MDPP may reduce pain-related disability, catastrophic thinking, reported degree of pain interference, and improve self-efficacy and chronic pain acceptance. Moreover, it may improve symptoms of depression and general psychological distress. There is some suggestion that these improvements are sustainable, being largely retained at 3-month follow-up. Given the increased time and commitment requirements of “Reboot Online” compared to other online pain management programs, the adherence rate is reasonable. This combined with the preliminary positive findings from the current pilot study suggest that “Reboot Online,” a comprehensive MDT online pain management program with a graded exercise component, warrants further investigation with a randomised controlled trial.

## Figures and Tables

**Table 1:** Participant demographic and pain characteristics.

	Number (20 in total)
Age	
Mean	47.45 years
Range	24–75 years

Sex	
Male	1
Female	19

Highest level of education	
Postgraduate	1
Undergraduate	8
Year 12	4
TAFE	2
Other certificate	4
No qualification	1

Employment Status	
Full time	3
Part time	2
Registered sick	6
Retired	4
At-home parent	3
Unemployed	2

Place of residence	
Major city	7
Inner regional area	3
Outer regional area	6
Remote area	4

Duration of chronic pain	
1-2 years	4
2–5 years	3
>5 years	13

**Table 2:** Estimated marginal means at pre-, mid-, and posttreatment and three months following “Reboot Online.”

	Pretreatment	Midtreatment	Posttreatment	3-month follow-up	Pre- to posttreatment			Pretreatment to follow-up		
	Mean (SD), *n*=20	Mean (SD), *n*=15	Mean (SD), *n*=14	Mean (SD), *n*=13	*F* (df)	Effect size: Hedges *g*	95% CI	*F* (df)	Effect size: Hedges *g*	95% CI
PSEQ	22.95 (13.77)	31.80 (13.79)	36.79 (13.77)	40.00 (13.77)	*F* (3, 58) = 13.84^*∗*^	−1.00	−1.72 to −0.28	*F* (3, 58) = 17.05^*∗∗*^	−1.24	−2.00 to −0.48
BPI-interference	7.26 (2.06)	5.27 (2.05)	4.25 (2.06)	4.11 (2.06)	*F* (3, 58) = 3.02^*∗∗*^	1.46	0.69–2.23	*F* (3, 58) = 3.15#	1.53	0.74 to 2.32
BPI-severity	5.75 (1.76)	4.71 (1.75)	4.27 (1.75)	3.69 (1.76)	*F* (3, 58) = 1.48	0.84	0.13–1.55	*F* (3, 58) = 2.06^*∗*^	1.17	0.42 to 1.92
PCS	24.50 (8.45)	19.00 (8.48)	13.33 (8.19)	13.55 (8.83)	*F* (3, 57) = 11.17^*∗∗*^	1.34	0.59–2.09	*F* (3, 57) = 10.96^*∗∗*^	1.27	0.51 to 2.03
TSK	40.05 (8.90)	35.47 (8.87)	34.14 (8.91)	34.18 (8.89)	*F* (3, 56) = 5.91	0.66	−0.04–1.36	*F* (3, 56) = 5.87	0.66	−0.06 to 1.38
PDI	43.60 (15.21)	3.72 (15.22)	31.21 (15.19)	27.55 (15.87)	*F* (3, 56) = 12.39	0.82	0.11–1.53	*F* (3, 56) = 16.06^*∗*^	1.03	0.29 to 1.77
CPAQ	51.95 (14.04)	63.73 (14.02)	69.50 (14.03)	70.18 (14.03)	*F* (3, 56) = 17.55^*∗∗*^	−1.25	−1.99 to −0.51	*F* (3, 56) = 18.23^*∗∗*^	−1.30	−2.07 to −0.53
DASS-D	17.10 (7.92)	11.87 (7.90)	8.57 (7.89)	8.91 (8.24)	*F* (3, 56) = 8.53^*∗*^	1.08	0.35–1.81	*F* (3, 56) = 8.19^*∗*^	1.01	0.27 to 1.75
DASS-A	11.10 (7.74)	9.47 (7.75)	6.29 (7.75)	5.09 (7.75)	*F* (3, 56) = 4.81	0.62	−0.08–1.32	*F* (3, 56) = 6.01	0.76	0.04 to 1.48
DASS-S	17.30 (9.21)	15.73 (9.22)	12.29 (9.24)	10.18 (9.63)	*F* (3, 56) = 5.01	0.54	−0.15–1.23	*F*(3, 56) = 7.12	0.76	0.04 to 1.48
K10	26.80 (6.57)	21.20 (6.58)	20.50 (6.59)	19.80 (6.93)	*F* (3, 55) = 6.30^*∗*^	0.96	0.24–1.68	*F* (3, 55) = 7.00^*∗*^	1.04	0.30 to 1.78
PHQ-9	12.10 (4.70)	9.40 (4.69)	8.29 (4.71)	7.18 (4.92)	*F* (3, 56) = 3.81	0.81	0.10–1.52	*F* (3, 56) = 4.92^*∗*^	1.02	0.28 to 1.76

^*∗*^ *p* < 0.05; ^*∗∗*^*p* < 0.01; #*p* < 0.001.

## Data Availability

The data used to support the findings of this study are available from the corresponding author upon request.
